# Trusted Energy-Aware Hierarchical Routing (TEAHR) for Wireless Sensor Networks

**DOI:** 10.3390/s25082519

**Published:** 2025-04-17

**Authors:** Vikas, Charu Wahi, Bharat Bhushan Sagar, Manisha Manjul

**Affiliations:** 1 Computer Science and Engineering, Birla Institute of Technology, Mesra 835215, India; charu@bitmesra.ac.in; 2 Computer Science and Engineering, Harcourt Butler Technical University, Kanpur 208002, India; bbsagar@hbtu.ac.in; 3 Computer Science and Engineering, Delhi Skill and Entrepreneurship University, Delhi 110077, India; manisha.manjul@dseu.ac.in

**Keywords:** wireless sensor network, trust management, energy, anomaly, routing

## Abstract

These days, wireless sensor networks (WSNs) are expanding fast and are used in many fields such as healthcare, battlefields, etc. Depending upon the type of sensor, they are transmitting a considerable amount of data in a short duration, so security is a significant issue while transferring the data. So, it is essential to solve security concerns while transferring data by secure routing in wireless sensor networks. We address this challenge by proposing Trusted Energy-Aware Hierarchical Routing (TEAHR), a new framework for a multi-level trust assessment that raises the security level in WSNs. TEAHR introduces a variety of trust metrics ranging from energy trust to forwarding trust to consistency trust to behavioral trust to anomaly detection, unlike existing models, enabling it to effectively address the challenges of dynamic network topologies and evolving cyber threats. Trust-based routing mechanisms are usually associated with high computation and storage complexity and susceptibility to collusive attacks such as spoofing. The mechanism in TEAHR overcomes these challenges by placing an adaptive trust assessment mechanism that adapts to the background network conditions and real-time activities of the nodes. We show through empirical analysis in this paper that TEAHR not only uses computational and storage resources efficiently but also enhances network performance and security. Our experimental setup presents the simulation approach to prove our proposed protocol of TEAHR in comparison with typical trust models under different scenarios of node mobility, variable node density, and sophisticated security attacks such as Sybil, wormhole, and replay attacks. TEAHR keeps the network connected, even when the nodes are isolated due to trust misbehavior, and demonstrates that widely it reduces the chances of misjudgment in trust evaluation. Moreover, we explore the scalability of TEAHR across large networks as well as its performance in computationally constrained contexts. We have verified through our detailed investigation that the energy metrics used uniquely in TEAHR extend the life of the network while increasing data routing trust and trustworthiness. The comparisons of TEAHR with conventional techniques show that the proposed algorithm reduces total latency by 15%, enhances energy efficiency by around 20%, and maintains a stable packet forwarding rate, which is highly desirable for accurate operation in adversarial environments, as demonstrated through comparative analysis. Through in-depth theoretical and practical analysis, TEAHR is confirmed as a high-performance framework that outperforms currently existing studies for WSN security, making TEAHR a strong candidate for use in industrial IoT applications and urban sensor networks.

## 1. Introduction

Trust, a fundamental component of human interactions, serves as a conceptual keystone for establishing secure and reliable communication among sensor nodes. In WSN, Trust assesses node behavior and encompasses a multifaceted evaluation of communication patterns, data integrity, and overall trustworthiness. Integrating trust models provides an upward route to defend WSNs from malicious features, generates fidelity of transmitted messages, and grows the life span of the network in operation. Abstract With the dramatic proliferation of wireless sensor networks, much is expected to provide a broad range of services and applications that come up with an overwhelming need for cost-effective, robust security solutions. Conventional WSNs, despite being irreplaceable in terms of their data-acquisition potential, are subject to limitations such as node compromise, which is tempered with data and communication interruptions. The vulnerabilities, escalated by the inherent resource constraints of sensor nodes, point towards a dire need for new ways to enhance the dependability and efficiency of wireless sensor networks. WSNs are naturally vulnerable because wireless communication is open, so we see many security threats. The concept of trust management for WSNs provides an easy and straightforward solution to couple the security and cooperation problem, which measures the degree/level by which sensor nodes in improving network performance are being trusted or distrusted. WSNs are capable of providing multi-tiered detection capability, as they can gather information wirelessly and communicate. However, there are also security flaws in their open messaging, unattended deployment, and limited resources. Malicious nodes (compromised or faulty) can inject erroneous data, disrupt routing protocols, and consume energy, thereby jeopardizing the network integrity and application performance.

Recent studies have proposed various trust evaluation models for assessing the trustworthiness of sensor nodes. Z. Ye. et al. [1] have proposed a dynamic trust evaluation model (DTEM), considering several factors of communication, called communication trust, data storage, and processing demands for adaptively evaluating data flow quality, which is referred to here for DTEM as energy-aware reputation-based scheduling in CSPs. This model calculates direct Trust and evaluates indirect Trust using trusted third-party recommendations. The integration of direct and indirect Trust was achieved by assigning and combining dynamic weights. Trust-based security mechanisms involve the development of lightweight security protocols tailored to the resource-constrained environments of WSNs. These are trust-based cluster head election mechanisms, location-aware trust-based mechanisms for detecting and isolating compromised nodes, and scalable enforcement algorithms using many-to-one balanced evolutionary game theory. Mathematical models underpin these techniques and provide a theoretical foundation for trust management systems. Trust management systems are comprehensive frameworks that include the collection, storage, modeling, transferring, and decision-making of trust-related information [2]. They are designed to detect and defend against internal attacks such as DoS, bad-mouthing, and on-off attacks, among others. Trust models offer an appropriate mechanism for formally setting up and handling trust relationships among sensor nodes. The Trust of sensor nodes is evaluated in many dimensions, including data trust, behavior trust, comprehensive Trust, and historical trust [3]. Relay nodes are also subjected to trust evaluation to ensure the integrity of the data transmission. A trust list was maintained to keep track of the trustworthiness of nodes within the network. Trust in wireless sensor networks is always considered a broader concept than just the evaluation of single-node behavior. This refers to communication patterns, data correctness verification, and collaborative decision-making. Through the amalgamation of trust metrics, anomaly detection algorithms, and cryptographic techniques, a comprehensive trust-based framework has emerged, which is promising for significantly enhancing the security posture of wireless sensor networks.

In WSNs, trust management requires customization of applications as trust management (TM) is challenging owing to the nature of WSNs, deployment of sensor nodes, and how the dynamic characteristics of sensor nodes change over time. The deployed sensor nodes create numerous complications in TM [4]. Hostile surroundings can harm or physically impact a sensor node, causing it to function poorly. In addition, sensors are vulnerable to physical capture and temptation because of the distant and unsupervised nature of the environment. This results in a node behaving in an intelligent but incorrect manner, further complicating this issue. The network’s topology is dynamic, causing the neighborhood to vary dynamically. It is crucial to manufacture sensors at a low cost, which results in limitations in their computational power, communication range, and battery capacity. Therefore, in light of these aspects, it is essential to develop trust estimations (TEs) that are effective in terms of energy and processing resources to align with the limitations of sensor nodes while also ensuring that they are strong enough to fulfil the security needs of TM. Furthermore, exchanging trust values between nodes may be restricted because of the increased energy consumption and congestion in the limited bandwidth caused by the message overhead. Proposing a trademark while considering the aforementioned considerations can be challenging. Executing TE and managing each Node in a large number of neighboring nodes is a tedious task. Despite numerous proposed studies, current computational techniques need a comprehensive analysis of all essential design factors for TM. Furthermore, while TM is still in its early stages, numerous research difficulties require attention, particularly the confusion between TM, reputation management, and trust establishment.

To ensure secure communication in WSNs, it is essential to ensure that the intermediate nodes responsible for forwarding data packets are trustworthy within the network. Hence, an effective trust model needs to be created. Each sensor node should evaluate the trustworthiness of its neighboring nodes based on model parameters. The trust model is one of the critical issues in WSNs. Various studies have also shown that a trust management system is a beneficial approach to identification and protection from threats [5]. Moreover, to avoid captured/compromised nodes from internal attacks, the trust model used here, the author has designed a trust management system. Trust management systems are employed to assess the attributes of information, provide network trustworthiness services such as authentication and sensing the rogue nodes, and facilitate secure resource sharing. This is a more profound research area in understanding Trust Management technology mechanisms, designs, and objectives of Trust management for WSNs, along with related study and open challenges. To find Trust, we need to cover the fundamental concepts of Trust, i.e., ideas and design elements. To construct a Trust Establishment (TE) scheme appropriately, it is crucial to have a thorough understanding of the fundamentals, i.e., the definition, values, and properties of Trust. Furthermore, TM-related attacks, protection methods, and applications are discussed to provide a comprehensive understanding of TM. Understanding the concept, values, and features of Trust is crucial for estimating the trust levels of nodes. The trust model establishes and manages trust connections between two nodes to ensure that legitimate nodes may be trusted to participate in the information transmission process [2]. Trust is the level of belief in the future actions of other nodes as determined by prior experiences and observations of their behavior. Trust in WSNs is as follows: The Trust that Node A has in Node B can be defined as the confidence, expectation, or assurance that Node B will be good in a manner that is sincere, competent, and honest in an upcoming activity or behavior. Trust value: A wide variety of forms and ranges are used, which can be in the range of 0 or 1, 0 to 100; the form can be discrete or continuous. Trust properties: Trust properties are yet another essential component of both trust estimation and establishment. We consider the following trust properties: the subjective attribute indicates that multiple trust values can be assigned to the same Node. The current degree of Trust may be influenced by past experiences. Continually changing leads to the possibility that trust values increase or decrease over time. The evaluation of Trust is influenced by the researcher’s subjective viewpoint, and there is no consensus on the specific traits that should be included. In previous studies, the authors in [6] categorized trust as Trust relationships built on context attributes that encompass information about the relevant participant’s situation. It is challenging to assess and monitor trust characteristics. These traits are related to cognitive and social Trust. Trust factors that may be assessed and tracked. These qualities focus on computing Trust. The authors [7] have defined trust qualities into numerous attributes, such as Trust is not 100%, i.e., one object cannot trust another object completely. The degree of Trust between nodes R and V and between nodes R and G is related to the transferability of Trust. This crucial quality can be used to reason through complex relationships. In the past, researchers have proposed various trust management approaches for WSNs, each with its strengths and limitations. WSNs are increasingly becoming a critical component of modern technology infrastructure. WSNs have evolved into a fundamental technology for collecting and transmitting data in a variety of settings. However, the decentralized and often unattended nature of these networks makes them particularly vulnerable to security threats. There are many challenges, and trust management is essential for ensuring the trustworthiness, security, and integrity of the data and the network itself. The deployment of WSNs in open, often hostile environments poses unique challenges that differ from traditional wired or centralized networks. The critical characteristics of WSNs include limited power resources, constrained computational capabilities, and the absence of a fixed infrastructure. These characteristics make WSNs highly susceptible to a range of attacks, such as data tampering, eavesdropping, and node capture. Furthermore, the ad hoc nature of these networks means that nodes must often rely on each other for routing data, making the network’s overall security dependent on the trustworthiness of individual nodes.

The motivation for selecting trust management in WSNs is also driven by the need to choose an approach that aligns with the specific requirements of the network and its applications. In trust-based routing protocols, the trustworthiness of nodes is considered when selecting routes for data transmission; considering this approach helps ensure that data are transmitted through reliable nodes, thus reducing the risk of data being intercepted or tampered with. Another approach is reputation systems, in which nodes monitor the behavior of their neighbors and assign reputation scores based on their observed behavior. Nodes with low reputation scores are considered untrustworthy and may be excluded from participating in the network. When you need to identify malicious behavior, you have to consider context-aware trust management systems, which are useful in dynamic environments where the behavior of nodes may vary depending on external factors such as environmental conditions or the presence of interference. By considering the context, these systems can make more accurate trust assessments. So, for WSNs, trust management is an essential focus for researchers and practitioners alike. By selecting trust management, WSNs can achieve higher levels of security, resilience, and efficiency, enabling them to support a wide range of critical applications across different domains. Trust is a dynamic concept, and it is possible for it to exist for some time. It is inevitable that the level of confidence shifts over time. WSNs are assailable to several types of attacks. Therefore, it is necessary to create a robust and secure design and use secure algorithms. This paper is further divided in the following ways: in Section 2, we discuss related studies. In Section 3, we focus on the proposed study and the discussion; Section 4 discusses the results, and Section 5 is the conclusion of the paper.

## 2. Related Studies

**Figure 1:**
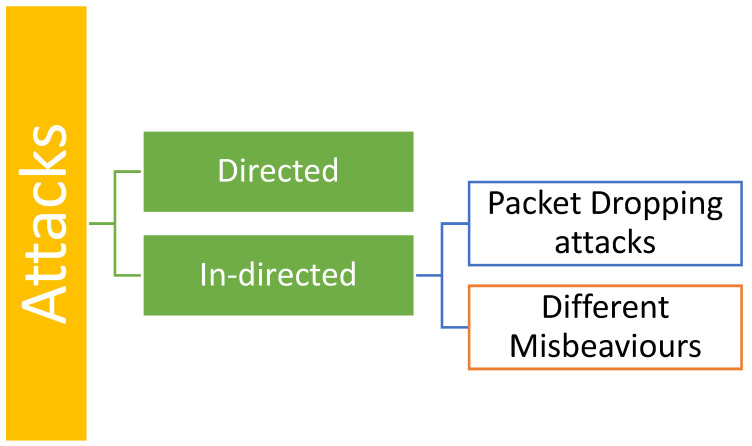
Attack in WSN.

As security is one of the major concerns apart from energy, data transfer, unreliable communication, etc. Figure 1 displays the kind of attack in WSN using TM. We can classify the attacks in two ways, i.e., direct and indirect. In direct attacks, the targeted attacks on the TM system result in a decline in TM system performance and cause inaccurate decision-making. Hence, the attack diminishes the efficiency of the network. In the context of secure routing, if the Traffic Manager (TM) is unable to identify hostile nodes owing to on-off or bad-mouthing assaults effectively, these malicious nodes can be chosen as routing nodes for packet transmission. Therefore, the network experienced an elevated number of dropped packets. TM can be customized using abundant parameters, such as TE interval, rewarding and punishing processes, trust threshold, forgetting factor, dynamic trust parameter changes, and trust aggregation parameters. The optimal configuration of a Traffic Manager (TM) is contingent upon the specific use of TM, prevailing security conditions inside the network, and intended design objectives. Nevertheless, a TM system must meet specific fundamental criteria, including its ability to withstand rudimentary attacks. The purpose of trust management is to identify and protect against malicious node attacks to strengthen network security. For instance, if a malevolent node fails to transmit received data, the level of Trust diminishes.

In indirect attacks, the assaults do not specifically target the TM system, but the TM system can detect and prevent them. Such attacks may include deliberate data manipulation, depletion of energy resources, disruption of time synchronization, inaccurate reporting of sensor data, and several other malevolent behaviors. Addressing such threats and malicious behavior are the primary goals of TM systems. A defense system against these attacks adheres to a standard process. Node performance was regularly monitored, observed, and documented. The defense procedure is mostly adjusted when employed to address various kinds of attacks. These attacks can be further classified as packet-dropping attacks in which malicious nodes attempt to impair the network operation by intentionally discarding packets using various methods, such as blackhole attacks, grayhole attacks, and wormhole attacks. Another type is different misbehaviors. In general, nodes that misbehave can be categorized into the following two types: selfish nodes, which prioritize their own gain even if they harm others, and malevolent nodes, which intentionally undermine the performance of the system or other nodes without seeking personal benefits. Attacks can vary depending on the intentions of the misbehaving nodes. If a malicious node wants to deplete the energy of other nodes, it sends a substantial volume of traffic and forces the other nodes to respond. Another instance involves the transmission of forged, modified, or repeated routing information with the intention of causing routing loops, manipulating network traffic, altering source routes, generating false error messages, dividing the network, increasing the time it takes for data to travel from one end to another, and so forth. Owing to these attacks, the trust value was impacted.

### 2.1. Types of Attacks

A malicious node can be identified by detecting its trust value in a timely manner. The comprehensive breakdown of the attacks is as follows:

- Bad-mouthing attack [8]: This attack involves malicious nodes attempting to undermine the credibility of trustworthy nodes or enhance the credibility of malicious nodes through the dissemination of dishonest suggestions.
- Ballot stuffing attack [2]: A Confederate node enhances its reputation by supplying a substantial quantity of successful interaction data to the other side. To address such assaults, it is essential to decrease the weight of the indirect trust value offered by the neighboring Node.
- On-off attack [9,10]: In an on-off assault, malicious nodes exhibit intermittent performance, alternating between periods of satisfactory and unsatisfactory behavior. Malicious nodes can retain trust values even if they exhibit subpar performance. To effectively counter-switching attacks, older behavioral observations must carry a different level of significance than the more current behavioral observations.
- Selfish attack [11]: The self-node will only delete the consent without reserving the resources to send the trust reply upon receipt of the trust request.
- Sybil attack [12]: The utilization of ID authentication and centralized trust models is a viable strategy to protect against Sybil attacks. These approaches not only enable the accurate identification of nodes but it also facilitate the detection of many false identities associated with malicious nodes through the network sink node or base station (BS).
- Sinkhole attack [13]: The assailant establishes a deceptive aggregation node to divert all data within the vicinity of the fraudulent sink node.
- Reputation time-varying attack [14]: A time-varying attack is a cyberattack strategy that changes its characteristics over time to evade detection and bypass security measures. These attacks exploit the vulnerabilities in systems designed to detect static attack patterns. By constantly changing their tactics, time-varying attacks can remain undetected for more extended periods, potentially causing more damage.

### 2.2. Trust Models and Their Classification

**Figure 2:**
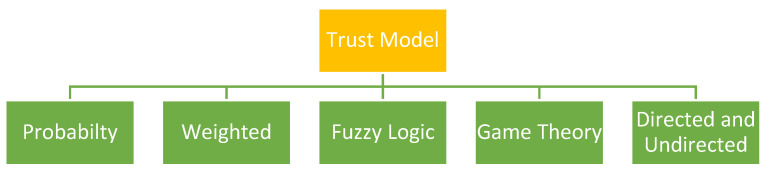
Trust model [12].

Recently, numerous schemes for trust management and reputation have been proposed, such as e-commerce, web-based services, peer-to-peer networks, and WSNs. It is essential to conduct a review of the most cutting-edge technology (TM) schemes that have been offered for WSNs to gain an understanding of the current status of the field and outstanding research concerns. In Figure 2, we present some proposed taxonomy schemes, comparisons, and TM schemes.

The probability trust model features are designed for security and trustworthiness in WSNs. Trust models help in assessing the trustworthiness of the sensor nodes and the data they transmit, which is crucial in environments where sensor readings are used to make critical decisions. They rely on probability theory to estimate the trustworthiness of each Node in the network, including parameters such as its previous behavior, recommendations from other nodes, and data provision quality. It is assumed that the sensor nodes will perform peer trust monitoring of each other, which represents a typical mode in practice for this problem under a probabilistic trust model. In a probabilistic trust model, which characterizes the usual approach to the issue in which each sensor node has its own monitoring trust score, it is expected that these sensors will behave or function correctly. This score is updated with time as the Node interacts with the network and the rest of the other nodes. The trust scores, if appropriately managed, can be used to decide whether a node is good enough for use in critical tasks or not, and if yes, then what type of queries should be answered by that particular Node while excluding others from the network based on their low trust levels. The resource-constrained nature of sensor nodes (e.g., power, memory, and computational capacity) is one of the most challenging barriers to developing trust models in WSNs. Thus, trust models should be light and efficient when employed on WSNs. This kind of probabilistic trust model is beneficial in situations where we have to make decisions on their behavior or the surroundings under uncertainties. It has a probabilistic strategy, and this makes the models capable of providing some estimation for Trust throughout the network, which is also vital while creating more secure WSNs. In WSNs, trust management typically relies on probabilistic models that use sensors’ behavior and interactions with other nodes in order to estimate the probability of the credibility for every Node. R. Feng et al. [15] proposed a trust model based on the Bayesian and entropy trust models. The Bayesian trust model updates the value based on the behavior of observed nodes and utilizes Bayesian inference.

(1) $$T_{new}=\frac{T_{old}\times P+S}{T_{old}+N}$$

$T_{Anew}$ is the new trust value; $T_{old}$ is the old trust value; P is the prior trust value; and S is the number of successful interactions. N is the total number of interactions. If we want to express the uncertainty in the Trust of some node, then this will be entropy. Therefore, lower entropy on the confidence of a node indicates that it is more reliable. The Trustfulness of a node, using Equation (2): The entropy (H) is defined based on the trust value in each Node as follows:

(2) $$H=-\sum_{i=1}^{n}p_{i}{\log x}_{2}p_{i}$$

where $p_{i}$ is the probability of the *i*-th outcome, and n is the number of possible outcomes. WSNs warrant a trust management regime to ensure that the information collected and disseminated by nodes is accurate and reliable. A weighted trust model in WSN is used to evaluate and govern a node’s credibility, which reserves its activities and interaction. The most appropriate model for this version is one that consists of components, each rated according to core and complementary networks. Trust metrics, trust evaluation, trust update, and trust-based decision-making are the significant components of the weighting trust model. Trust metrics are characteristics that indicate the trustworthiness of a sensor node that is resourceful. These may be the packet forwarding ratio, data trustworthiness, energy level, and cooperation degree per node, where each quality indicator is weighted with an essential factor for Trust to calculate. In the probability trust model, we also need to consider the direct and indirect Trust. The direct observations and recommendations are used to calculate direct and indirect Trust. Overall, Trust (T) can be a weighted sum of the direct $T_{d}$ and indirect $T_{i}$ as follows:

(3) $$T=w_{d}\times T_{d}+w_{i}\times T_{i}$$

$T_{Wd}$ is direct weight, and $w_{i}$ is indirect weight. As in WSN, where the trusts aggregate from different sources, and finally, the trust level of the recommenders is considered in the weighted average:

(4) $$T_{agg}=\frac{\sum_{j=1}^{m}w_{j}\times T_{j}}{\sum_{j=1}^{m}w_{j}}$$

$T_{agg}$ is the aggregated trust value, $T_{j}$ is the trust value from the *j*-th recommender, $w_{j}$ is the weight assigned to the *j*-th recommender’s trust value, and m is the number of recommenders. A simple approach is to model the trust score T of a node using a weighted-sum model.

(5) $$T=\sum_{i=1}^{n}w_{i}\times m_{i}$$

where $w_{i}$ is the weight of the *i*-th metrics and $m_{i}$ is the value of the *i*-th metrics. In order to track the relevance of trust scores, tools like Trust Update mechanisms are highly required. Trust scores can adapt by making updates in their values on new interactions or behaviors and developing methods to forgive nodes as well as penalize based on the change of actions. Trust scores are used in certain network decisions, such as routing and data aggregation or node cooperation (these will be discussed later). Nodes with a high trust score should be given priority for particular jobs, whereas those that are untrusted might end up being quarantined or scrutinized further. C Wahi et al. [16] Trust-Oriented Routing in Mobile Ad Hoc Networks (MANET) proposed a trust-based routing framework for establishing routes with neighboring nodes, increasing AODV security levels by using historical node data to calculate the cooperation level, including both direct and indirect recommendations, and recompensing cross-recommendations. It is confirmed by a sequence of tests that such a version improved over TSAODV, called original AODV and TSR, across all scenarios in this study, but significantly when different parameters regarding the total number of nodes and distribution node velocity are changed. Research on it strengthens the weighted trust model and uses Machine Learning Algorithms (MLAs) that update weights depending on the network state, so this adaptivity improves the accuracy of trust evaluation. A crucial illustrative example was found in a series of studies by Alshehri et al. These advances are discussed by [17], which showed better network performance and security. Authors Chen et al. Present behavior trust, data trust, and Historical Trust. The comprehensive Trust is calculated through a weighted approach, where each type of Trust contributes to the overall trustworthiness of a node. The formula for comprehensive Trust could be represented as *comprehensives = w*_1_ × *T_behavior_+ w*_2_ × *T_data_ + w*_3_ × *T_historical_, where w*_1_, *w*_2_, and *w*_3_ are the weights assigned to each trust component, respectively. Reddy et al. [18] have introduced a model that evaluates both communication trust and data trust. Communication trust is derived from direct and indirect observations of a node’s forwarding behavior, while data trust is computed using the median of sensor data. The direct Trust could be calculated as follows:

(6) $$T_{direct}=\frac{successful\;forward}{\;total\;forwards}$$

Moreover, the Indirect Trust uses the weighted Dempster–Shaffer theory. The authors Prabha et al. [19] employed fuzzy logic to evaluate Trust by considering metrics such as message success rate, elapsed time at a node, correctness, and fairness. Ye et al. [1] proposed a dynamic trust evaluation model (DTEM) that dynamically adjusted the weights of direct and indirect Trust. Direct Trust includes communication, data, and energy trust, whereas indirect Trust is derived from trusted recommendations. The integrated Trust can be calculated as follows:

(7) $$T_{integrated}=\alpha \;\times \;T_{direct}+\left(1-\alpha \right)\;\times \;T_{indirect}$$

where α is the weighted factor dynamically adjusted based on network conditions. Prabha et al. [19] proposed a trust model based on fuzzy logic to assess the trustworthiness of nodes in WSNs by considering several metrics as follows: the message success rate, elapsed time at a node, correctness, and fairness. The trust value in this model is calculated by the fuzzification of these input metrics and the application of fuzzy inference rules. Finally, defuzzification of the result is used to find the final trust level, which is usually further broken down into low, medium, or high. Although not explicitly stated in the paper, a typical step in most fuzzy logic systems is fuzzification, in which all crisp input values are changed into degrees of membership of fuzzy sets. For example, the message success rate can be placed under sets such as ‘Low’, ‘Medium’, and ‘High’. The trust value can be calculated using the membership function μ, as follows:

(8) $$T=\frac{\mu_{High}\left(x\right)}{High}+\frac{\mu_{Medium}\left(x\right)}{Medium}+\frac{\mu_{Low}\left(x\right)}{Low}$$

where x is an input parameter, such as the number of successful data transmissions by M. Patra et al. [20]. A fuzzy inference rule in which IF-THEN rules are applied based on fuzzified inputs to determine the trust level. An example rule might be IF (Message Success Rate is High) AND (Elapsed Time is Low) THEN (Trust is High). The defuzzification converts the fuzzy output of the inference process into a crisp value that represents the final trust level of the Node.

(9) $$Y_{crisp}=\frac{\sum_{i=1}^{n}\mu_{Y}\left(y_{i}\right)\;\times \;y_{i}}{\sum_{i=1}^{n}\mu_{Y}\left(y_{i}\right)}$$

where $\mu_{Y}\left(y_{i}\right)$ denotes the membership value of the output variable at $y_{i}$ D. Han et al. [21]. In trust aggregation, the trust values from different sources can be aggregated using fuzzy operators such as the fuzzy weighted average as follows:

(10) $$T_{agg}=\frac{\sum_{i=1}^{k}w_{i}\;\times \;T_{i}}{\sum_{i=1}^{k}w_{i}}$$

where $T_{i}$ is the trust value from the *i*-th source, and *w_i_* is the weight assigned to it by R. Patel et al. [22]. The overall Trust value T can be conceptualized as a function of the input metrics processed through the fuzzy logic system as follows: T = *f* (Message Success Rate, Elapsed Time, Correctness, Fairness). The Game theory-based trust models in WSNs apply the principles of game theory to analyze and optimize interactions among sensor nodes, focusing on Trust. These models treat interactions as strategic games in which nodes make decisions on their objectives, constraints, and expected actions by other nodes. The objective is to design mechanisms that promote cooperative behavior while discouraging the behavior of malicious or selfish nodes, thereby improving the overall trustworthiness of the network. In such a trust model, under game theory, every sensor node represents a player. The available strategies for players include forwarding packets, participating in network operations, and conserving energy. The results from these strategies, owing to the actions of other nodes, determine trust levels and, thus, payoffs for the players. In principle, Trust can be modeled as a game by using a trust model.

(11) Γ=(N,S,u)

where N is the set of players (sensor nodes), S = S_1_ × S_2_ × …… × S_n_ is the set of strategy profiles, where $s_{i}$ is the strategy set available for player I; u = u_1_ × u_2_ × … × u_n_ is the vector of payoff functions, with $u_{i}$ (s_1_ × s_2_ × … × s_n_) representing the payoff to player i when strategy profile (s_1_ × s_2_ × …… × s_n_) is employed. The level of Trust of a node can be derived from its payoff, which reflects the behavior of this Node to achieve network objectives, such as good data transmission. The common approach to modeling this uses utility functions that capture the benefits and costs related to different strategies. A simplified utility function by Abdalzaher et al. [23] for a sensor node may be written as follows:

(12) $$u_{i}(s_{i},s-i)=\alpha \times B(s_{i},s-i)-\beta \times C(s_{i})$$

where $u_{i}(s_{i},s-i)$ is the utility for Node I choosing strategy $s_{i}$ is given strategies *s* − *i* of other nodes; $B(s_{i},s-i)$ represents the benefits obtained from choosing strategy $s_{i}$, which may depend on the actions of other nodes. C($s_{i}$) for costs incurred if strategy si is chosen, α and β are weighting factors that maintain a balance between the benefits and costs. Those who define game models often look for Nash equilibria where none of the players feel compelled to change their strategy independently, being convinced of the strategies of all the others. In this context, regarding Trust, a Nash Equilibrium by Abdalzaher et al. [24] outlines the situation in which all nodes act in such a way as to maximize their utility while maintaining a satisfactory degree of trustworthiness and cooperativeness. Most related studies in this area have focused on developing sophisticated mechanisms for dynamically adaptive changes in networks. At the same time, it has been incorporated with realistic factors, such as node mobility, variable trust levels, and possible adversaries. An evolutionary game is the study of the strategy evolution dynamics in a population. M.Adnan et al. [25] proposed a WSNs model of how trust-related strategies evolve among sensor nodes, and for that, the replicator equation is defined as follows:

(13) $${\dot{x}}_{i}=x_{i}\left(U_{i}\left(x\right)-U^{-}\left(x\right)\right)$$

where ${\dot{x}}_{i}$ is the proportion of the population using strategy I, $U_{i}\left(x\right)$ is the rate of change of x_i_, $U_{i}\left(x\right)$ is the utility of strategy I, and U(x)) is the average utility of the population. The models of Trust in Wireless Sensor Networks (WSNs) are the directed graph-based trust model and the undirected graph-based trust model. In both cases, graph theory is utilized to show relationships and interactions among the sensor nodes, where levels of Trust are shown in the graph’s edges. The nodes and relationships represent the vertices defined by the edges within these models. Directed Graphs, or simply Digraphs, are used when the trust relationship is said to be asymmetric. That is, the trustworthiness of A to B is not necessarily the same as that of B to A. Moreover, in undirected graphs, symmetric trust relationships were presented. That is, Trust is mutual and equal in both directions. In directed graph-based models, each edge is associated with a given direction. Directed edges show the relation of Trust from one Node to another, usually labelled with a weight. Then, the edge may further be used to represent the level of confidence. Such a model could use the sum of all incoming edge weights as a measure of the trustworthiness of the Node:

(14) T(v)=∑(u,v)∈Ew(u,v)

Here, T(v) is the trust value for mode v, E is the set of edges, and w(u,v) refers to the weight of the edge from node u to node v. In undirected graph-based models, the edges are not directed and represent the mutual trust relationship between the nodes. The level of Trust, in this case, can be calculated based on the average weight of the edges that it connects as follows:

(15) T(v)=deg(v)1∑(v,u)∈Ew(v,u)

where deg(v) is the degree of node v, and w(v,u) is the weight of the edge connecting nodes v and u. This graph can also support extra features, such as history data, node behavior, and social trust metrics. For instance, a dynamic weighted graph model may change the edge weights with time, according to node actions, following mathematical expressions, to gradually increase or decrease the level of Trust from positive or negative interaction. Also, some of the most recent studies have been using sophisticated techniques, such as machine learning, for predictions and updating trust levels in conjunction with graph-based models. For example, the study conducted by Wang et al. [26] presented a dynamic model of trust management based on the directed graph, where temporal and spatial parameters are taken into account to change the trust levels depending on network conditions and behaviors. In his study, Xinying Yu et al. [27] have established the Energy Trust Model (ETM), which includes the remaining energy of nodes and trust level to obtain secure communication paths that will consequently guarantee the confidentiality of transmitting data within a WSN.

### 2.3. Hierarchical Routing

A large number of routing protocols have been proposed in WSN because of their inherent characteristics, as well as their application and architectural requirements. These routing protocols can be categorized based on the network structure, protocol operation, and path base. The selection of an appropriate hierarchical routing algorithm should consider various factors like network size, topology, traffic patterns, and energy constraints. These are essential factors that need to be explicitly considered while choosing an algorithm for any application in WSN.

**Figure 3:**
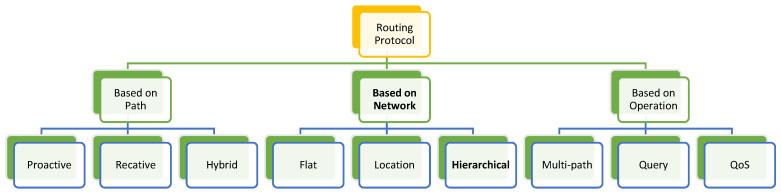
Routing protocols in WSN [27,28].

Figure 3 displays the routing protocol for the WSN and in above diagram the hierarchical routing algorithm in WSNs is essential for reducing energy consumption and improving the transmission efficiency of large-scale deployments as well. Hierarchical algorithms arrange the nodes in a hierarchy and then partition the network into clusters or partitions. In the cluster, most packets are forwarded through direct communication with CH or trusted intermediate nodes if it is not possible to communicate directly. Inter-cluster Communication means communication between clusters. When communicating outside the cluster, CH relays packets to other local CHs or a base station. This will allow routing decisions based on Trust to firmly find the paths for both trustworthiness and energy efficiency. Nodes whose trust levels fall below the minimum Threshold due to malicious actions or inconsistent behavior can be isolated from critical network operations or removed from the network to maintain overall security and efficiency. By systematically managing Trust and continuously monitoring the behavior of both existing and new cluster members, a WSN can effectively safeguard against malicious nodes (anomaly) and ensure reliable and secure network operation. Here are a few hierarchical routing algorithms that can be found in virtually all WSNs.

LEACH [29] (Low-Energy Adaptive Clustering Hierarchy) is a system in which nodes select the CH based on the probability threshold. Therefore, CHs aggregate data from their member nodes and transmit this to a sink node. LEACH is scalable and very easy to deploy, but it may need more energy consumption across nodes, especially near the sink. Power Efficient Gathering in Sensor Information Systems (PEGASIS) [30] is a token-passing mechanism in which each sensor node receives from and transmits to the nearest sensor node, and CH, having a token, will transmit it to BS; by doing so, it conserves energy. The token is rotated to make sure all sensor nodes participate in routing. PEGASIS is sensitive to node failure, especially CH. In this protocol, nodes closer to the sink node may consume more energy due to a more significant increase in transmission. Ad-hoc On-Demand Distance Vector Routing (AODV) [31] is not required in this global occurrence of advertising at specific intervals. Overall bandwidth is less required by each sensor node in this routing protocol. Apart from these, researchers have proposed many more routing algorithms.

### 2.4. Problem with Existing Schemes

Trust-based algorithms have many inherent problems that can impact their performance, trustworthiness, and overall effectiveness. While various trust models have been proposed for Wireless Sensor Networks (WSNs), they suffer from inherent limitations that impact their performance and trustworthiness. Key limitations include poor scalability in large networks, high energy overhead on tiny battery-powered nodes, susceptibility to certain malicious tactics, and inflexibility in dynamic environments. Limited scalability is a major shortcoming of many early trust models. As WSN deployments grow to hundreds or thousands of nodes, the overhead of maintaining and exchanging trust information explodes. Each node may need to monitor and store trust values for numerous neighbors and broadcast updates, which does not scale well. In large networks, this can **saturate communication channels and memory**, slowing down the whole system. Authors [32] note that a WSN with a “large number of sensor nodes” requires a **scalable algorithm** for trust management, prompting the use of clustering to handle many nodes efficiently. In flat (non-hierarchical) trust schemes, adding more nodes exponentially increases messages and computations, making them impractical beyond small network sizes. A specific failure example is a simulated 1000-node WSN using a flat trust table is as follows: the trust update traffic overwhelmed the network, delaying data delivery and even causing packet losses due to congestion (an implicit **scalability issue** that motivated hierarchical trust protocols). In summary, conventional models often **do not scale**—they may work in testbeds of 20 or 50 nodes but start to break down in large-scale deployments because of communication overhead and centralization bottlenecks. WSN nodes are **severely energy-constrained**, typically running on small batteries with limited capacity. A robust trust mechanism must be lightweight; otherwise, the energy cost of continuous monitoring and trust computation can shorten node lifespan dramatically. Many existing trust models did not fully account for this, leading to high energy drain. The **resource constraints (power, memory, CPU)** of sensors are “one of the most challenging barriers” in designing trust systems [10]. Frequent trust updates (e.g., broadcasting recommendations or heartbeats) and complex calculations (like Bayesian updates or cryptographic validations) consume extra energy on top of normal sensing duties. For instance, a node that must perform intensive trust calculations for every packet will use more CPU and radio time, accelerating its battery depletion. Beyond performance issues, many trust models are vulnerable to **sophisticated attacks** where malicious nodes exploit the trust system itself. Three notorious attack strategies in WSNs are **Sybil attacks**, **bad-mouthing attacks**, and **on-off (selective misbehavior) attacks**. In a Sybil attack, a single adversary forges many fake identities (pretending to be multiple distinct sensor nodes). WSNs frequently experience node join/leave events (due to node failures and deployments), so an attacker introducing new Sybil nodes “appears as new nodes” and is hard to immediately distinguish from legitimate joins [33]. Bad-mouthing (also called slander) involves malicious collusion to **spread false negative reputations** about honest nodes. In a reputation-based trust system, nodes often rely on peer recommendations; a group of compromised nodes can exploit this by all reporting a target node as untrustworthy (even if it behaved correctly). An experiment comparing trust frameworks showed that a model without strong collusion defense (referred to as LDTS) performed poorly against bad-mouthing—it was rated “Low” in security against such attacks [10]. In an on-off attack, a malicious node alternates between good behavior and bad behavior in cycles. The idea is to **exploit the trust update dynamics**—the node behaves well (“on”) just long enough to raise its trust score above a threshold; then, when sufficiently trusted, it behaves maliciously (“off”) for a period (e.g., dropping packets or injecting false data), then switches back to good behavior before its trust drops too low. Researchers [9] found that without special handling, such on-off attackers can continually exploit the system; they proposed a scheme to discriminate between “disguised malicious behaviors” and genuine temporary errors.

## 3. Proposed Studies

In WSNs, identifying compromised or malicious nodes that can disrupt data flow or infuse harm is a sensor network, and in order to evaluate the trust, the trust mechanism can be used. In this paper, we propose a Trusted Energy-Aware Hierarchical Routing (TEAHR) protocol, which is capable of addressing the following issues that WSNs have to encounter, such as limited energy, network security, and scalability. Sensor nodes are equipped with tiny batteries, so energy management must be taken care of cautiously. WSNs are threatened by many attacks, and trust mechanisms can resist malicious behavior. TEAHR protocols can efficiently cope with large-scale WSNs. Table 1 summarizes the above various types of trust management in WSN.

### 3.1. Research Contribution

In this paper, we proposed an energy-efficient framework to enhance the wireless sensor network security and detect anomaly detection. The TEAHR framework is designed to improve the security and performance of WSNs by dynamically assessing the trustworthiness of sensor nodes based on multiple criteria. The core idea behind TEAHR is to integrate trust evaluation with energy-aware routing to create a robust and efficient network. In the proposed framework, by considering factors such as energy consumption, packet forwarding effectiveness, consistency in behavior, and adherence to network protocols, the TEAHR framework provides a comprehensive approach to managing Trust and enhancing the trustworthiness of WSNs. The critical components of TEAHR are as follows:

- Energy Trust: Energy trust refers to the evaluation of a node’s remaining energy levels as a measure of its operational trustworthiness and expected lifespan. In WSNs, energy efficiency plays a critical role due to the limited power resources of sensor nodes, which are often powered by tiny batteries. Nodes with higher energy reserves are more likely to continue functioning reliably, making them more trustworthy within the network.
- Forwarding Trust: Forwarding Trust assesses a node’s effectiveness in forwarding packets to their intended destination. In a WSN, the ability of a node to reliably forward data is crucial for maintaining data integrity and ensuring that information reaches the intended recipients. A node’s forwarding Trust is determined by monitoring its packet forwarding history, including metrics such as the number of successfully forwarded packets, packet loss rates, and transmission delays. Nodes that demonstrate consistency and trustworthiness in forwarding behavior are assigned higher trust scores, which make them more favorable for inclusion in routing paths.
- Consistency Trust: The level of consistency in a node’s behavior over time, particularly in relation to packet forwarding, is evaluated by consistency trust. This metric helps detect deviations that may signal potential malignancy or hardware failure. Inconsistencies in a node’s behavior, such as sudden drops in forwarding success rates or erratic energy consumption, can indicate that the Node has been compromised or is experiencing technical issues. TEAHR can identify nodes that may pose a risk to the network and adjust routing decisions accordingly to maintain overall network stability.
- Behavioral Trust: Behavioral Trust examines a node’s adherence to network protocols and its past activity patterns to detect anomalies or potential security breaches. This metric is critical for identifying nodes that may be engaging in malicious activities, such as data tampering, unauthorized access, or collusion with other compromised nodes. Behavioral Trust is assessed by analyzing a node’s interaction with other nodes, its response to protocol commands, and any deviations from expected behavior. Nodes that exhibit suspicious or non-compliant behavior are assigned lower trust scores and may be excluded from critical network functions.
- Anomaly Detection: a vital feature of the TEAHR framework, enabling the identification and isolation of malicious nodes within the network. When a node’s trust score falls below a certain threshold due to suspicious behavior, it is flagged as potentially compromised. The TEAHR system then takes proactive measures to manage the anomaly, such as restricting the Node’s access to the network or isolating it entirely. This approach ensures that the impact of malicious nodes is minimized and that the network remains secure and operational. Additionally, the system continuously monitors the trust scores of all nodes, allowing for dynamic adjustments based on real-time behavior.
- Energy Efficiency: TEAHR improves energy efficiency across the network by using energy metrics in trust computation. Nodes that are more energy-efficient are rated positively in trust evaluations, which incentivizes the use of energy-saving practices among nodes. This focus on energy efficiency not only extends the operational life of the network but also enhances its overall performance by reducing the likelihood of node failures due to energy depletion.

Advantages of the Proposed TEAHR Framework. The TEAHR framework offers several advantages over traditional trust management and routing protocols in WSNs:

- Enhanced Security: By incorporating multiple trust metrics, including energy, forwarding, consistency, and behavioral Trust, TEAHR provides a comprehensive approach to identifying and mitigating security threats. This multifaceted trust evaluation process makes it more difficult for malicious nodes to evade detection, thereby enhancing the overall security of the network.
- Optimized Energy Consumption: The integration of energy trust into the routing process ensures that nodes with sufficient energy reserves are prioritized, reducing the likelihood of network disruptions due to node failures. This focus on energy efficiency helps to extend the lifespan of the network and reduces the need for frequent maintenance or node replacement.
- Improved Network trustworthiness: TEAHR’s emphasis on forwarding trust and consistency trust ensures that data are transmitted through reliable and consistent nodes, which enhances the integrity of the data and reduces the likelihood of packet loss or delays. This improved trustworthiness is essential in critical applications where accurate and timely data transmission is essential.
- Scalability: The hierarchical nature of the TEAHR framework allows it to efficiently manage trust and routing decisions in large-scale WSNs. The system can scale to accommodate networks with a large number of nodes without compromising on performance or security, making it suitable for deployment in diverse and expansive environments.
- Dynamic Trust Management: TEAHR’s ability to dynamically assess and adjust trust scores based on real-time behavior ensures that the network can quickly respond to emerging threats and changes in node behavior. This adaptability is crucial in environments where network conditions and node behavior can change rapidly, such as in battlefield or disaster response scenarios.

**Figure 4:**
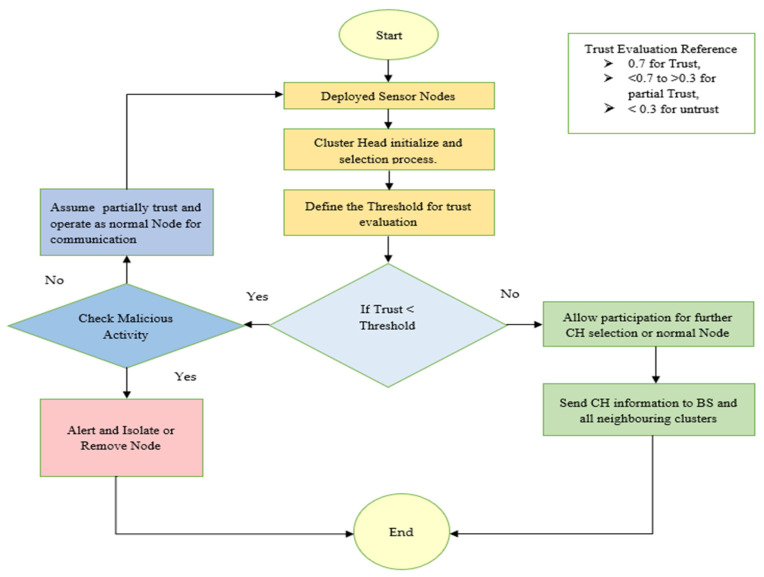
Flowchart of TEAHR.

The proposed TEAHR framework represents a significant advancement in the field of WSNs, offering a robust and energy-efficient solution for trust management and routing. By integrating multiple trust metrics and focusing on energy efficiency, TEAHR addresses the critical challenges of security, trustworthiness, and scalability in WSNs. The framework’s ability to dynamically assess Trust and manage anomalies ensures that the network remains secure and operational, even in the face of evolving threats. As WSNs continue to expand and find applications in critical areas, the TEAHR framework provides a valuable tool for enhancing network performance and security. Figure 4 shows the flow of the proposed algorithm.

In the proposed TEAHR framework, we deploy the sensor nodes in a 1000 × 1000 m area and start the cluster head (CH) initialization and selection process. To choose the CH, we define the threshold value (which is defined above; check the Trust Evaluation Reference) based on the energy level of each sensor node. If the trust level is less than the Threshold, then check for malicious activities. If the sensor node is a malicious (anomaly) node, then discard it; otherwise, participate in sending, receiving, and forwarding the data to CH. If the Trust is more than the Threshold, then use those sensor nodes to select the CH for data transfer activities.

### 3.2. Proposed Algorithm

As per the definition of Trust, it is not easy to create Trust in the dynamic nature of wireless sensor networks. In our proposed study, we tried to create Trust between sensor-to-sensor nodes and sensor nodes to cluster heads, as well as between the cluster heads. So, on the basis of this Trust, we measure the anomaly in the wireless sensor network, and we propose a framework for that. In the proposed algorithm, it is designed to evaluate the trustworthiness of nodes within a WSN. The variables used in this algorithm are presented next. xi: Represents an individual standard (non-cluster head) Node within the network. CH: Represents a cluster-head node. Cluster heads are specific nodes that take on additional responsibilities, such as coordinating communication within their cluster. CH.T: This is the Trust Table associated with a Cluster Head. It stores trust-related information about the normal nodes in that cluster. On the basis of these variables, the Trust is calculated as follows: T_energy(x_i_): Energy Trust. Measures how much energy a node has remaining relative to its maximum capacity (E_res(x_i_)/E_max). Nodes running low on energy might be more susceptible to compromise. T_forward(x_i_): Forwarding Trust. Calculates how reliably a node forwards data packets as intended (P_forwarded(x_i_)/P_received(x_i_)). T_consistency(x_i_): Consistency Trust.

Ensures that nodes are forwarding data packets consistently (P_consistent(x_i_)/P_forwarded(x_i_)). Inconsistent behavior could indicate problems. T_behavior(x_i_): Behavior Trust. A more complex measure is based on whether the Node follows the network rules and its past activity. So, overall, Trust and Threshold T_overall(x_i_): Combined trust score for the Node, calculated using a weighted sum of individual trust factors (with weights w1, w2, w3, and w4). T_threshold: Dynamically adjusted Threshold based on the network’s current state and past data. This allows the algorithm to adapt to changing conditions. The algorithm of the proposed study is as follows.

In Algorithm 1 the lines 1–2 Initialization of cluster_head_selection, which takes the network N and cluster head CH. Loop through each node x_i_ in the cluster of CH. Lines 3–9 If x_i_ is in the CH.T trust table, calculate the trust metrics, i.e., Energy Trust, Forwarding Trust, Consistency Trust, and Behavior Trust. Then, compute the overall trust score T_overall(x_i_)T_overall(x_i_)T_overall(x_i_) based on the weighted sum of the individual trust metrics. Then, a dynamic trust threshold T will be set based on network conditions and historical data. Lines 10–21 will perform the decision to find the Trust if the overall trust score T_overall(x_i_) is below the Threshold: If x_i_ shows signs of malicious behavior, then broadcast an alert about x_i_ and block x_i_ from all network activities. Otherwise, Flag x_i_ for re-evaluation to limit its network participation and update the trust level of x_i_ in the trust table CH.T. If the overall trust score T_overall(x_i_) is above the Threshold, then confirm x_i_ participation in network activities and update the trust level of x_i_ in the trust table CH.T. Line 22 End the loop for each Node in the cluster.
**Algorithm 1**: Cluster Head Selection**Input**: x_i_ represents the normal Node, and CH represents a Cluster-Head node. **Output**: Cluster head selection 1:  function cluster_head_selection(N, CH) 2:         for each x_i_ in CH’s cluster do 3:                 if xi in CH.T then 4:                          T_energy(x_i_) ⇐ E_res(x_i_)/E_max 5:                          T_forward(x_i_) ⇐ P_forwarded(x_i_)/P_received(x_i_) 6:             $T\_consistency(x_{i})\;⇐\;P\_consistent(x_{i})/P\_forwarded(x_{i})$ 7:                          T_behavior(x_i_) ⇐ evaluate_behavior(x_i_) 8:                              T_overall(x_i_) ⇐ w1*T_energy(x_i_) + w2*T_forward(x_i_) + w3*T_consistency(x_i_) + w4*T_behavior(x_i_) 9:                          T_threshold ⇐ set_dynamic_trust_threshold() 10:                         if T_overall(x_i_) < T_threshold then 11:                                if is_malicious(x_i_) then 12:                                      broadcast_alert(x_i_) 13:                                      block_node(x_i_) 14:                                else 15:                                      flag_for_re_evaluation(x_i_) 16:                                       update_trust_level(CH.T, x_i_) 17:                                end if 18:                         else 19:                                confirm_participation(x_i_) 20:                                update_trust_level(CH.T, x_i_) 21:                         end if 22:                 end if 23:         end for 24: end function

In Algorithm 2 the lines 1–5 Define the function setup_dynamic_trust_thresholds, which takes the network N and cluster head CH. For each node xi in the cluster of CH, calculate the initial trust threshold T_threshold and continuously update it. Lines 6–17 evaluate the Trust for all nodes, the same as in previous algorithm with additional adjusted routes dynamically based on the latest trust assessments. If the overall trust score T_overall(x_i_) is below the threshold T_threshold, redirect critical communications away from x_i_. Lines 19–29: If a new member is added for each new Nodenode in network N if the new Nodenode meets or exceeds the trust threshold T_threshold, fully integrate the Nodenode into the network with standard privileges. If the new Node fails to meet the trust threshold T_threshold: If probation is needed, extend the probation period for the Node. Otherwise, the Node can be removed from the network based on security policies. Lines 30–34 Periodically recalculate the trust thresholds. Recalculate T_threshold during significant network events, such as node failures or attack detection. Use adaptive algorithms to set thresholds based on predictive analytics of network conditions and node behavior patterns. Lines 35–42 manage the malicious behavior; if the overall trust score falls below T_threshold and the Node shows signs of malicious behavior, then isolate and remove xi from critical communication routes and broadcast alerts. Line 43 is the end of the loop.
**Algorithm 2**: Setup Dynamic Trust Thresholds**Input**: xi represents a normal node, and CH denotes a cluster-head node managing a cluster of nodes. **Output**: Dynamic threshold value 1: function setup_dynamic_trust_thresholds(N, CH) 2:         for each x_i_ in CH’s cluster do 3:                 T_threshold ⇐ calculate_initial_threshold(x_i_) 4: continuously_update_threshold(T_threshold) 5: end for 6:         for each x_i_ in N do 7:                 if x_i_ in CH.T then 8:                          T_energy(x_i_) ⇐ E_res(x_i_)/E_max 9:                          T_forward(x_i_) ⇐ P_forwarded(x_i_)/P_received(x_i_) 10: T_consistency(x_i_) P_consistent(x_i_)/P_forwarded(x_i_) 11:                          T_behavior(x_i_) ⇐ evaluate_behavior(x_i_) 12:                          T_overall(x_i_) ⇐ w1*T_energy(x_i_) + w2*T_forward(x_i_) + w3*T_consistency(x_i_) + w4*T_behavior(x_i_) 13: adjust_routes_based_on_trust(T_overall(x_i_), T_threshold) 14:                          if T_overall(x_i_) < T_threshold then 15:                               redirect_critical_communications(xi) 16:                          end if 17:                 end if 18:         end for 19:         for each new_node in N do 20:                 if new_node meets_or_exceeds T_threshold then 21: integrate_node(new_node) 22:                 else 23:                          if probation_needed(new_node) then 24:                               extend_probation(new_node) 25:                          else 26:                               remove_node(new_node) 27:                          end if 28:                 end if 29:         end for 30:         periodically_recalculate_thresholds() 31: for each significant_event in network_events do 32:                 recalculate_T_threshold() 33:                 set_thresholds_using_predictive_analytics() 34: end for 35:         for each x_i_ in N do 36: if T_overall(x_i_) < T_threshold and is_malicious(x_i_), then 37:                          isolate_and_remove(x_i_) 38:                          broadcast_alerts(x_i_) 39:                          quarantine_node(x_i_) 40:                 end if 41:                 update_CH_T() 42:         end for 43: end function

Algorithm 3 is Trust-based hierarchical routing oversees the routing process through trust assessments and energy factors. This method invokes method 1 (cluster_head_selection(N and CH)) to pick cluster heads based on Trust and employs the dynamic trust thresholds established by just previous algorithm for routing decisions. Establishes a hierarchical routing mechanism that is energy-efficient and trust-centric, guaranteeing adequate and secure data transmission across the network. In this algorithm, trust-based routing, data transmission, dynamic trust management, and secure data transmission functions are used to create hierarchical routing.
**Algorithm 3:** Trust-Based Hierarchical Routing**Input:** N—Set of normal nodes, CH—Set of Cluster Heads, BS—Base Station **Output:** Efficient and secure routing of data based on Trust 1: function initialize_network(N, CH, and BS) 2:         Deploy N sensor nodes randomly in the network area 3:         Divide N into M clusters using a clustering algorithm 4:         Select initial CHs using cluster_head_selection(N and CH) 5: Initialize trust levels for all nodes in N and CHs in CH 6:         Set up initial routing tables for each Node and CH 7: end function 8: function trust_based_routing_setup(N, CH, and BS) 9:         for each node x_i_ in N do 10:                 Identify the closest Cluster Head CH_i_ based on minimum energy cost 11:                 if T_overall(x_i_) ≥ T_threshold then 12:                          Assign node x_i_ to cluster CH_i_ 13:                          Update routing table of xi to CH_i_ 14:                 else 15:                          Flag x_i_ for re-evaluation and assign to probationary CH 16:                 end if 17:         end for 18: end function 19: function trust_based_data_transmission(N, CH, and BS) 20:         for each Cluster Head CH_i_ in CH do 21:                 Aggregate data from all nodes in CH_i_’s cluster 22:                 Calculate Chi _Trust based on T_overall values of nodes in the cluster 23:                 if CH_i__trust >= T_threshold then 24:                          Transmit aggregated data to BS using shortest path routing 25:                 else 26:                          Redirect data transmission to secondary or neighboring CH 27:                          Trigger cluster_head_selection(N and CH) for re-election of CH 28:                 end if 29:         end for 30: end function 31: function dynamic_trust_management(N, CH, and BS) 32:         for each node x_i_ in N do 33:                 Recalculate T_overall(x_i_) using updated parameters 34:                 Adjust cluster membership based on T_overall(x_i_) 35:                 if T_overall(x_i_) < T_threshold then 36:                          Trigger re-evaluation or isolate xi based on severity 37:                          Update routing tables accordingly 38:                 end if 39:         end for 40:         Update dynamic trust thresholds using setup_dynamic_trust_thresholds(N and CH) 41:         Adjust routes based on updated trust values and thresholds 42: end function 43: function secure_data_transmission(N, CH, and BS) 44:         for each Cluster Head CH_i_ in CH do 45:                 Verify T_overall(CH_i_) and establish a secure communication link 46:                 Transmit data to BS via trusted CHs or multi-hop routing if required 47:                 if link failure or malicious behavior is detected, then 48:                          Isolate and reroute data through alternative CH or path 49:                 end if 50:         end for 51:         Periodically monitor the network for any malicious(anomaly) activities 52: end function 53: function maintain_network(N, CH, and BS) 54:         for each node x_i_ in N do 55:                 Monitor energy levels and T_overall(x_i_) 56:                 Re-elect CHs based on residual energy and trust levels using cluster_head_selection(N, CH) 57:         end for 58:         for each Cluster Head CHi in CH do 59:                 Evaluate performance and reassign nodes if necessary 60:         end for 61:         Perform periodic network maintenance and recalibrate trust parameters 62: end function

#### 3.2.1. Theoretical Analysis of TEAHR

##### Formal Proof of TEAHR’s Robustness in Trust Evaluation

**Theorem 1.**  *TEAHR is Robust Against anomaly Sensor Nodes in Trust Estimation.* 

**Proof**  **by Strong Induction.** 

Let T(x, y, Δt) denote the trust value assigned by sensor node x to sensor node y at time Δt.

Base Case: No Interaction Between Nodes x and y. If there is no direct interaction between x and y, the trust value T(x, y, Δt) is computed indirectly by the Cluster Head (CH), which aggregates recommendations from neighboring nodes. Since CHs are periodically verified and their trust values are aggregated using weighted averaging, malicious nodes cannot manipulate indirect trust values arbitrarily. TEAHR remains robust even when there are no direct interactions. Inductive Hypothesis (For k Interactions). Assume TEAHR correctly evaluates trust and prevents deception for all cases where the number of interactions ≤ k. Inductive Step (For k+1 Interactions). If the number of successful interactions S(x, y, Δt) is greater than the unsuccessful interactions U(x, y, Δt), then T(x, y, Δt) remains above the trust threshold (T_thresh), allowing normal participation. If U(x, y, Δt) > S(x, y, Δt), TEAHR detects the node as potentially anomaly detection to detect sudden fluctuations in trust scores. Thus, by induction, TEAHR remains robust for all k+1 interactions, proving that no node can manipulate the trust model without detection. □

**Theorem 2.**  *TEAHR is Robust Against Anomaly Cluster Heads (CHs) in Hierarchical Trust Evaluations.* 

**Proof**  **by Contradiction.** 

Suppose TEAHR is not robust, meaning an anomaly CH can manipulate trust scores. Each CH maintains a local trust table (LTT) and exchanges trust metrics with neighboring CHs. If a CH misbehaves (e.g., dropping packets, falsifying reports, or colluding with malicious nodes), TEAHR enforces the cross-verification with other CHs: if CH-A detects that CH-B is lowering trust scores unjustifiably, CH-B is penalized. Adaptive Trust Thresholds: CHs dynamically update the T_thresh, preventing attackers from exploiting static trust models. Anomaly Isolation: If a CH’s behavior deviates significantly, it is flagged, demoted, or isolated. Thus, no CH can manipulate trust scores without being detected, leading to a contradiction. □

##### Complexity Analysis of TEAHR

Computational Complexity

- Trust Score Computation: Each node computes its trust score using a weighted sum of metrics (Energy Trust, Forwarding Trust, Consistency Trust, Behavioral Trust). Time Complexity: O(1) per node.
- Cluster Head Selection: CHs are selected based on energy levels and trust scores. Worst-case Complexity: O(N log N) (sorting-based selection).
- Anomaly Detection:
  ○ Sliding window-based detection: O(1) per node.
  ○ Statistical deviation-based anomaly detection: O(N) time.

Communication Complexity

- Trust Exchange (Node-to-CH): O(1) per node, O(N) per cluster.
- CH-to-CH Trust Verification: O(log N) per CH.
- Global Network Trust Aggregation (Base Station processing): O(log N).

Thus, TEAHR achieves O(N log N) worst-case complexity, making it scalable for large WSN deployments.

##### TEAHR Under Adversarial Conditions

i. Cluster Member-to-Cluster Member (CM-to-CM) Trust Estimation

- No Interaction (CM x and CM y):
  ○ Ux,y C, D (Δt) + Sx,y C, D (Δt) = 0 → Trust is computed by CH, ensuring robustness.
- If Ux,y C, D (Δt) ≥ 1 and Sx,y C, D (Δt) = 0:
  ○ TEAHR assigns Tx,y C, D (Δt) = 0, preventing malicious deception.
- If Ux,y C, D (Δt) ≥ Sx,y C, D (Δt):
  ○ Trust score Tx,y C, D (Δt) < Θ, flagging the node as malicious.

ii. Prevention of Common WSN Attacks

1. Sybil Attack Prevention
  ○ TEAHR enforces ID authentication and anomaly detection.
  ○ Nodes with multiple IDs are flagged when behavioral discrepancies are detected.
2. On-Off Attack Defense
  ○ TEAHR introduces trust decay factors.
  ○ Nodes alternating between good and bad behavior face exponential trust score decay, preventing cyclic exploitation.
3. Bad-Mouthing and Ballot-Stuffing Attack Mitigation
  ○ TEAHR cross-verifies recommendations from multiple sources.
  ○ Weighted averaging of indirect trust prevents a group of malicious nodes from unfairly penalizing others.
4. Blackhole and Wormhole Attack Prevention
  ○ TEAHR flags nodes dropping packets at an abnormally high rate.
  ○ If a CH fails multiple integrity checks, it is demoted, isolated, and replaced.

**Figure 5:**
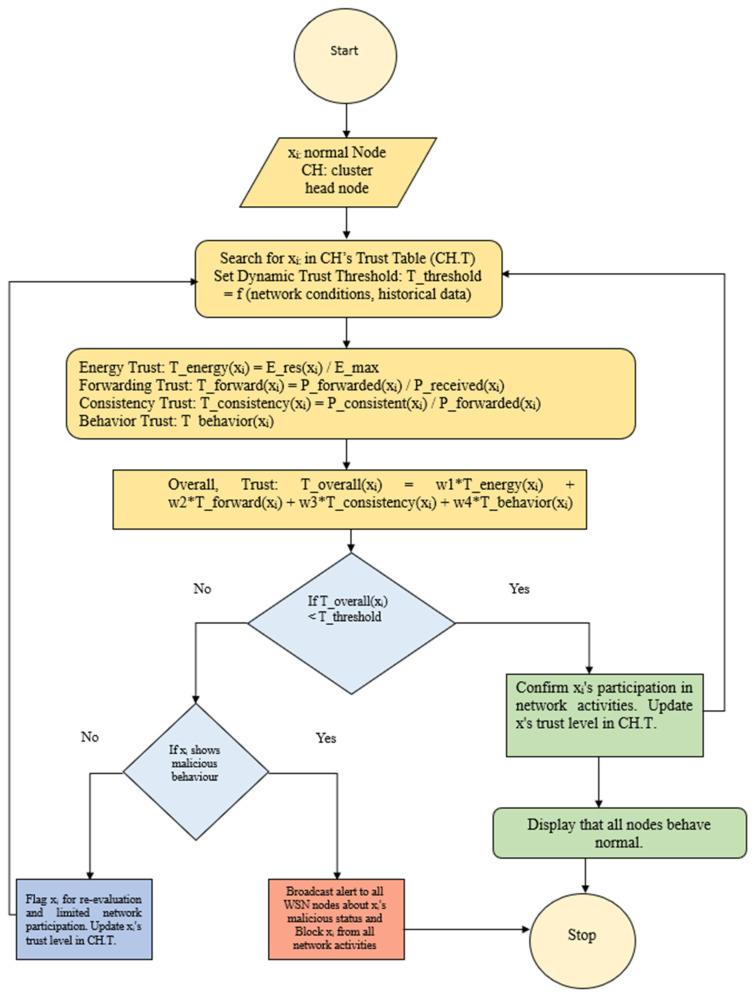
**The** flow of CH selection.

In Figure 5 shows a dynamic trust evaluation framework for WSN to detect a malicious node and isolate that node. Each node *x_i_* is evaluated by its cluster head (CH) based on four metrics i.e., energy trust, forwarding trust, consistency trust and behaviors trust. These trusts are combined using the weighted to calculate overall trust, which is being compared with dynamic threshold value. If trust value meets or exceed the threshold value then that sensor node be consider trustworthiness and continue allow them in network. If it is failed below the threshold but not shows malicious behaviors then allow them to be part of network or if shows the malicious behaviors then discard those sensor nodes. These steps will ensure the security and by continuous monitoring the node behaviors. 

## 4. Result and Discussion

### 4.1. Trusted Energy Aware Hierarchical Routing (TEAHR)

Authors [34] demonstrate the effectiveness of the TIOCHR algorithm in enhancing the network lifetime and energy efficiency; it needs to fully explore the potential challenges of implementing such trust-based systems in highly dynamic or adversarial environments where node trustworthiness might frequently change. A dynamically changing WSN experiences node failure, mobility, energy depletion, and varying network traffic. To maintain robustness, TEAHR’s trust assessment mechanism must be adaptive across multiple trust metrics. Trust scores are updated continuously, monitoring nodes’ actions. If a node’s behavior fluctuates (e.g., it forwards packets inconsistently), its trust score is gradually lowered. Nodes with stable, positive behavior receive incremental trust increases. If a node starts dropping packets due to congestion, the system distinguishes unintentional failures from malicious intent by analyzing past behavior. Instead of immediately isolating the node, TEAHR might reduce trust slowly and allow recovery if normal behavior resumes. Furthermore, this study might benefit from a comparative analysis with other trust-based models to establish the specific advantages or limitations of the TIOCHR approach in various real-world applications. In our proposed model, we tried to overcome the drawback of TIOCHR.

**Figure 6:**
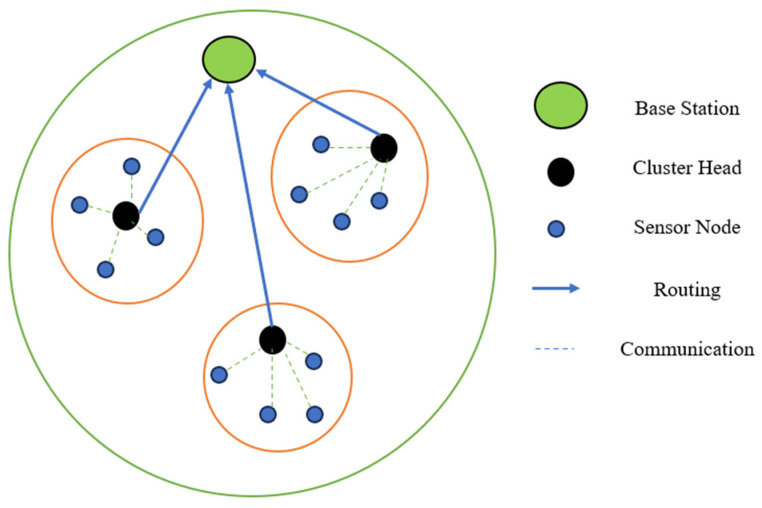
Sensor-to-sensor, sensor-to-group head, cluster head to cluster head-to-group head, and cluster head to base station trust and routing.

In Figure 6 green color is base station which gathers all information of the sensor network, black color is cluster head of which is surrounded by the sensor nodes are blue color. Bule line shows that routing from cluster head to based station and dotted line shows the sensor node communication to cluster head.

Table 2 shows the notations descriptions for various trust and selection metrics used in WSN for cluster-based architecture. The parameters such as energy, communication, node behavior and other network related factors to find the trustworthiness.

### 4.2. Initialization

Each Node in the network starts with an initial percentage of being a cluster head (CH) candidate, called the “CH probability” (P_ch_), and sets its status to undecided. The initial P_ch_ can be a system parameter based on the size and density. Let P_ch_(i) be the probability of Node i becoming a CH. Each Node I broadcasts its P_ch_(i) along with its residual energy, E_res_(i). The nodes assess their residual energy relative to a reference, which can be the initial energy or an average energy level. This step ensures that nodes with higher energy have a higher chance of becoming a cluster head. Let E_res_(i) be the residual energy of Node i, and E_ref_ be the reference energy level, which could be the initial energy level E_init_ or the average energy level E_avg_ of all the nodes in the network. The fundamental trust score T_basic_ (i) of Node i can be estimated as follows based on the residual energy of Node i:

(16) $$T_{basic}(i)=E_{res}(i)/E_{ref}$$

This equation means that the trust score is directly proportional to the lasting energy of a node compared with the chosen reference energy level. In a more dynamic scenario, where it becomes relevant that a chosen maximum residual energy, E_max_, for the network at the time of evaluation could change over time, the normalized trust score would be:

(17) $$T_{norm}(i)=E_{res}(i)/E_{max}$$

The normalization of the trust score is such that it falls within a range [0, 1], with one corresponding to a node with maximum residual energy in the network. Furthermore, F(i) can have several other factors related to the centrality of the Node, its mobility, or historical performance in trust assessment. Then, a weighted trust score $T_{weighted}(i)$ can be in the form:

(18) $$T_{weighted}(i)=w_{1}(E_{res}(i)/E_{ref})+w_{2}F(i)$$

where $w_{1}$ and $w_{2}$ are the weights assigned to the energy-based trust component and the additional factors, respectively, with $w_{1}$ + $w_{2}$ = 1. For Adaptive CH Probability Update each non-CH candidate node j, the updated CH probability P′_ch_ (j) is calculated as follows:

(19) $$P_{ch}^{\prime }(j)=P_{ch}(j)+\alpha (E_{res}(j)/E_{avg\_ann}-1)$$

Here, $E_{avg\_ann}$ is the average of the residual energies announced by neighboring nodes, and α is a scaling factor to adjust the influence of the residual energy difference. So on the basis of that, the tentative CH selection Evaluation for each non-CH node k evaluates tentative CHs based on a suitability metric S(i,k), which could consider the announced Eres and Pch, and selects the one with the highest S(i,k).

(20) $$S\left(i,k\right)=\beta_{1}E_{res}(i)+\beta_{2}D(i,k)+\beta_{3}P_{ch}(i)$$

Here, D(i,k) represents the distance or signal strength between nodes i and k, and $\beta_{1},\;\beta_{2},\;{and\;\beta }_{3}$ are weights. Nodes with similar $E_{res}$ might be further evaluated based on secondary parameters like proximity or communication cost C(i,k), selecting the CH that minimizes this cost. Minimize C(i,k). The final CH set is determined by the nodes that receive confirmations from at least one other Node. Each non-CH Node joins the nearest or most suitable CH based on a composite metric M(i,j) considering Eres, D(i,j), and C(i,j).

(21) M(i,j)=γ1 Eres(j)−γ2D(i,j)−γ3C(i,j)

Here, γ1 γ2 γ3 are weights. The CH should continuously evaluate the trust values of the existing cluster members based on energy backup, packet forwarding, packet consistency, and any other relevant parameters. The network should define dynamic trust thresholds that can be adjusted over time based on network conditions and requirements. The initial trust $T_{threshold}$ can be set on historical data or network specifications.

(22) $$T_{threshold}=f(network\;conditions,historical\;data)$$

This $T_{hreshold}$ is adaptive, based on the network’s historical data and current conditions. Trust-based communication and packet forwarding routes within the cluster should be established based on trust values. Higher trust nodes should be preferred for critical roles, such as data aggregation or relay.

### 4.3. Integrating New Cluster Members

When a new node is added to the cluster, an initial trust assessment is conducted. New nodes start with an initial trust value based on standard network entry criteria, possibly lower than the trust threshold for existing members to account for the lack of historical behavior data. This could involve directly monitoring the Node’s behavior in terms of its packet forwarding rate, energy usage, and consistency of data provided.

(23) $$T_{new}(i,t)=T_{initial}+\delta (T_{observed\;actions}(i,t))$$

Here, $\delta (T_{observed\;actions}\left(i,t\right))$ is the trust changes with time t from when a new node joined based on its types of observation, such as packet forwarding behavior, energy consumption, and proper implementation of network protocols. As a first step, the initial trust threshold of new nodes can be set to slightly less than that for current members so they have time to put their performance where their mouth is. Nevertheless, this maximal Threshold should be enough to hinder obviously nefarious nodes from perturbing mission-critical roles. New nodes may serve a probationary term during which they are observed more carefully. The trust value of those peers is prone to change more often, and their network roles are limited to non-critical tasks until a steady-state level of high Trust has been achieved and security checks are performed to verify if the new Node did not arise from known attacks. This could mean checking the Node’s credentials, confirming that its software has not been tampered with, and examining whether it is communicating in an abnormal way. Other nodes in the network watch how new ones on board participate and gradually increase their Trust as it becomes clear they are providing a benefit to the operations of that network. As the trust level of a new node grows and exceeds the dynamic Threshold, it can then be assigned additional tasks (i.e., data aggregation or being a secondary relay) within the cluster.

In our proposed algorithm, hierarchical routing is used and focuses on selecting CH from the group of sensor nodes, which is responsible for maintaining the information about the nodes within its cluster, calculating the Trust and making decisions about whether the Node is trustworthy or malicious. Our proposed algorithm, TEAHR, calculates the trustworthiness of nodes based on energy, forwarding behavior and consistency and dynamically adjusts the Threshold to decide whether a node can participate in selecting CH or should be blocked. As mentioned, the proposed algorithm updates the trust threshold dynamically and recalculates it based on various network events, further emphasizing the hierarchical decision-making process at the CH level.

### 4.4. Simulation Environment

Table 3 defines the parameters used in this proposed model; in this table, we define the trustworthiness, packet forward rate, anomaly factor, energy, trust level, threshold for detecting the malicious Node (anomaly), etc.

### 4.5. Deployment of Sensor Nodes

**Figure 7:**
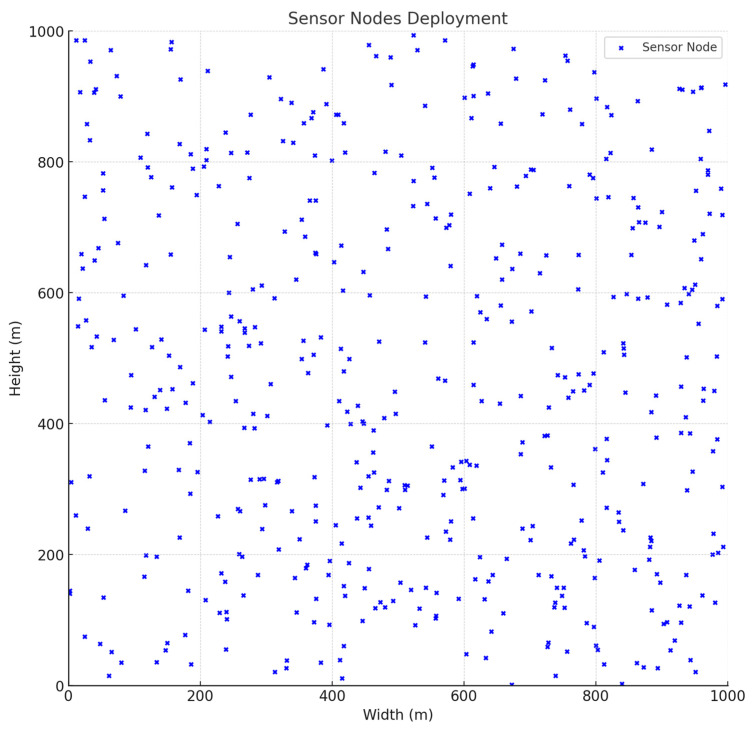
Sensor node deployments.

In this paper, we set up the environment using 1000 × 1000 m^2^, and 500 sensors are randomly distributed. Figure 7 shows the deployment of the sensor nodes. Trustworthiness (TB(x_i_)), Packet Forwarding Rate (PFR), Anomaly Factor (AF), Malicious Threshold (TB(x_i_)), Trust Evaluation Interval, and New Node Trust z are used to evaluate the proposed study. The initial energy for each sensor node is 100 J.

Cluster Head (CH) Selection: To select the cluster head (CH), the minimum threshold range is from 0 (untrustworthy) to 1 (fully trustworthy). The minimum trust level required for CH selection = 0.7; nodes with >50% of initial energy are eligible for CH selection = 0.5. Malicious Trust threshold = 0.3 will be considered a malicious Node, and the trust level required value is 0.7 for CH selection.

**Figure 8:**
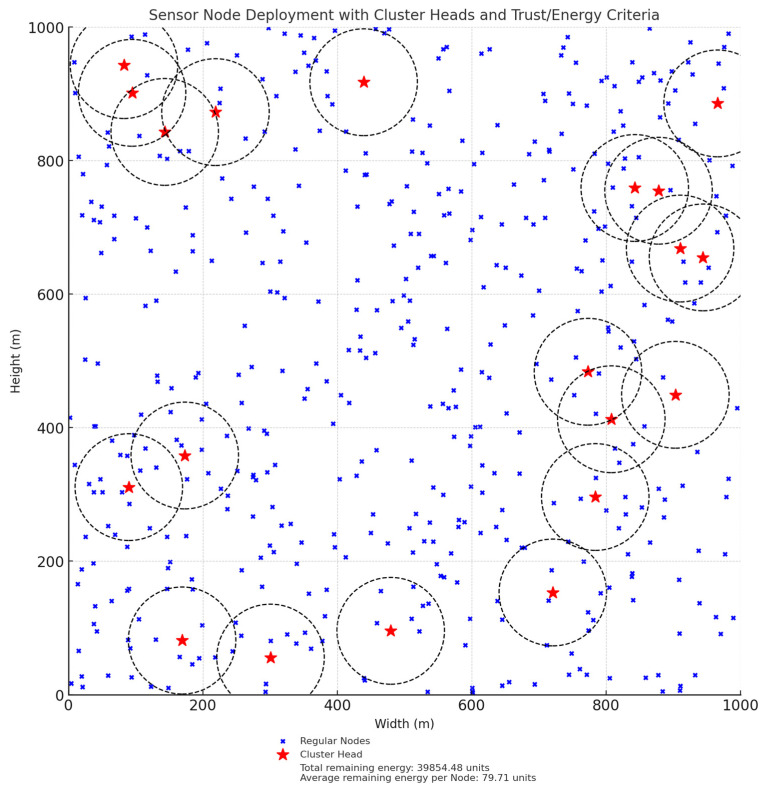
Cluster head selection.

Figure 8 displays the cluster heads chosen out of 500 sensor nodes on the basis of defined energy. Now, we insert the 20% malicious nodes to find the anomaly in the given sensor network as we insert the 20%, i.e., 100 malicious sensor nodes—the energy of overall sensor nodes after selecting the cluster head and identifying the malicious node. The dashed black circle around a CH represents the area within which sensor nodes communicate with each other via cluster head either directly or indirectly. 

**Figure 9:**
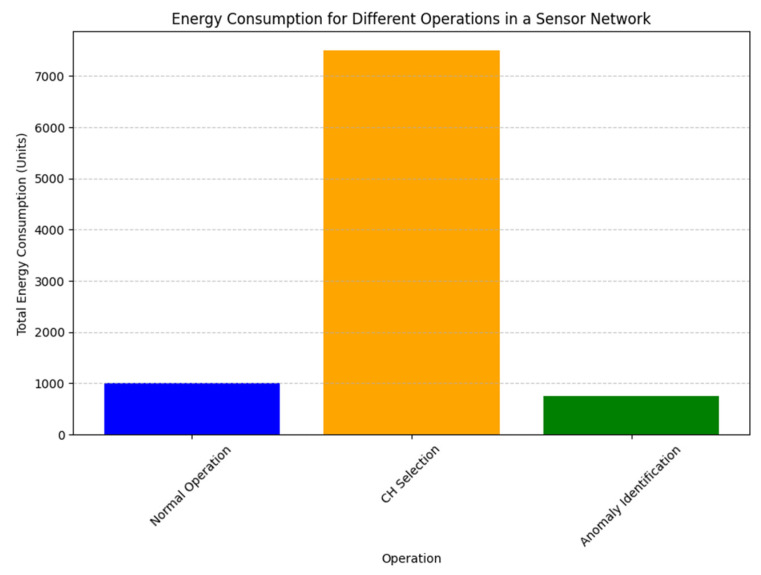
Energy consumption during normal operation, CH selection, and anomaly identification in WSN.

Initial energy for each node = 1.0, energy consumed for forwarding a packet = 0.01, Threshold below which nodes are blacklisted = 0.3, number of packets each Node attempts to send = 100. Figure 9 displays the total energy consumed by different operations in sensor network mainly three i.e., normal operation, CH selection and anomaly identification. For normal operation such as sensing and communication consume around 1000 units, while CH selection shows the highest energy consumption which is 7000 units and for anomaly identification energy consume approximately 800 unit. 

**Figure 10:**
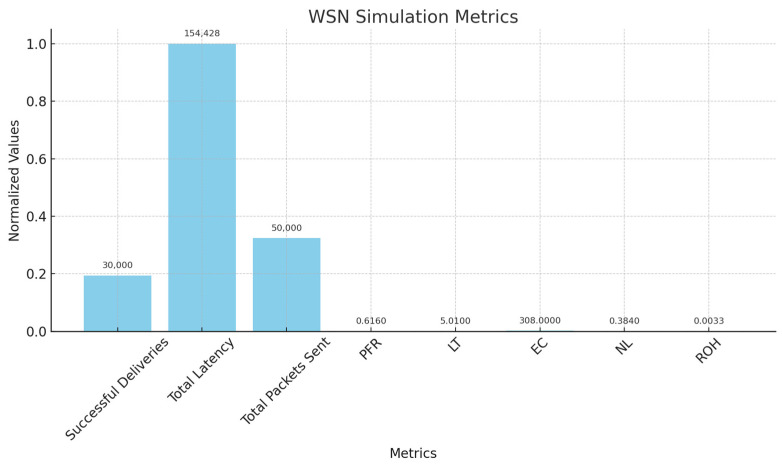
Successful deliveries, total latency, total packets sent, pfr, lt, ec, nl, and roh.

Figure 10 shows various performance indicators while WSN simulation. The metrics are displayed normalized values to compare across different scales. he successful deliveries = 30,000 represent the number of packets delivered, total latency = 154,428 reflects cumulative communication delay in the network, total packets sent = 50,000 shows the overall volume of data generated, packet forwarding rate (pfr) = 0.616 shows the reliability, latency (lt) = 5.013 is average delay per transmission, energy consumption (ec) = 308.00, network longevity as a fraction of alive nodes (NL) = 0.384 shows operation efficiency of the network over time, Routing Overhead (ROH) = 0.0033 shows protocol related overhead.

Identifying the suitable algorithm requires a balance between energy efficiency and latency, especially as the network grows. The graph below represents the trade-offs that different algorithms present when scaling up a network. TIOCHR and especially TEAHR appear to be more scalable solutions with respect to latency.

As defined in Algorithms 1 and 2, the TEAHR adopts a multi-level trust assessment mechanism, which includes energy trust, forwarding trust, consistency trust, behavioral trust, and anomaly detection. These may increase computation and storage overhead. To determine whether it does impose an additional performance burden or not, we have analyzed the performance impact of the multi-level assessment as defined in Algorithms 1 and 2, the result of which is Table 4, which shows that the trust assessment mechanism introduced a minimal additional overhead and the TEAHR approach is feasible without significant performance degradation.

The multi-level trust assessment mechanism helps in detecting and mitigating anomaly nodes, but it is vulnerable to strategic spoofing attacks where malicious nodes can be able to manipulate trust metrics to evade anomaly detection.

Table 5 provides a range of measures related to spoofing attacks and how these attacks affect network performance and integrity. num_nodes represents the total number of nodes in the network. num_anomaly represents the number of nodes that have been designated as anomalies. These are nodes suspected or confirmed to participate in spoofing or other harmful activities. anomaly_percentage defines the percentage of nodes that are malicious relative to the total number of nodes. This offers a normalized view of how prevalent anomaly activity is within the network. pre_attack_excluded count of nodes or connections that were excluded (or filtered out) by the network’s security mechanisms before any spoofing attack occurred. pre_attack_malicious_excluded nodes excluded before the attack, this column represents those that were malignant. It is an indication of the initial effectiveness of the pre-attack filtering mechanisms in catching malicious nodes. pre_attack_detection_rate is a percentage that illustrates the effectiveness of detection mechanisms before an attack. post_attack_excluded refers to the number of nodes (or connections) that were excluded after the spoofing attack was underway. post_attack_malicious_excluded the nodes after the attack begins; this column counts those that were identified as anomalies. post_attack_detection_rate is a detection rate computed after the spoofing attack begins. spoofing_success_rate metric indicates the success rate of the spoofing attack in terms of how many spoofing attempts or malicious actions were effectively carried out on the network. A higher value suggests that the attack was more successful in bypassing security measures.

Table 5 shows the spoofing attack analysis; in this we compare the spoofing attack in 500, 1000, and 2000 sensor nodes. The percentage of malicious nodes remains consistent across all sample sizes (approximately 29%), but the spoofing vulnerability increases slightly with the full dataset (48.21% compared to 47.26% for 1000 nodes and 42.07% for 500 nodes). This suggests that as the network scales, the percentage of malicious nodes that can successfully spoof the system increases, making larger networks potentially more vulnerable to strategic spoofing attacks. The data demonstrates that anomaly nodes can effectively execute strategic spoofing attacks against TEAHR, with high success rates and significant network compromise levels, particularly as networks scale up in size.

If some nodes are isolated due to misjudgment in WSN, they will be treated as anomaly nodes, and isolating them affects the network connectivity. If we drop those nodes, it leads to reduced global connectivity and might separate the network into many disconnected components. More nodes will be removed, and its size will decrease. The data flow, resiliency, and cooperation among nodes are affected by this. An increase in connectivity can drive down the average path length for small communities; however, homogeneities can form that can reduce end-to-end communications across the network. In general, we avoid connection of anomaly (malicious) nodes to the rest of the network. However, identifying benign nodes as anomalies led to unnecessary fragmentation and weakened the overall robustness of the network. If we reassess those misjudged isolated sensors, they may lead to overhead as it will increase the computation. In TEAHR, reintegration of wrongly isolated nodes should be considered if they were misclassified; for that, adaptive re-routing mechanisms should be optimized to ensure sustained packet delivery rates. Lastly, this further emphasizes the need to strike the right balance of node accuracy to maintain network connectivity while still preserving security. So, in future analysis could focus on the long-term impact on latency and routing overhead.

### 4.6. Analysis of TEAHR

**Figure 11:**
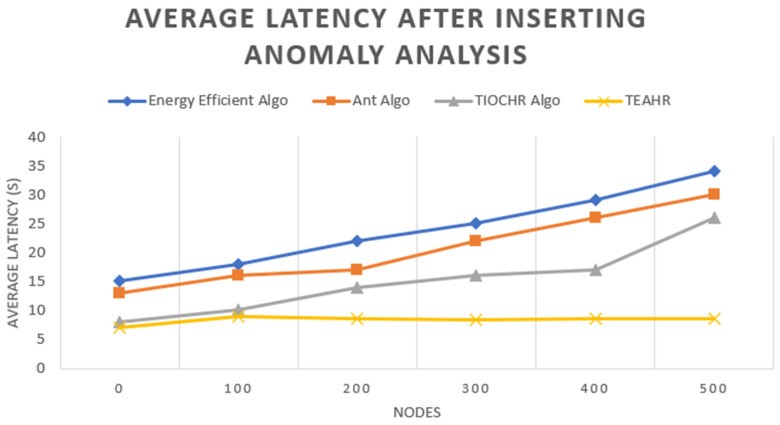
Average latency analysis Energy Efficient Algo [35], Ant Algo [36], and TIOCHR Alo [34].

In Figure 11, the proposed algorithm is compared with other algorithms, and the proposed algorithm reduces total latency by 15% after inserting the anomaly as defined above. As shown in Figure 11, the average latency of the TEAHR algorithm is more stable than other algorithms.

**Figure 12:**
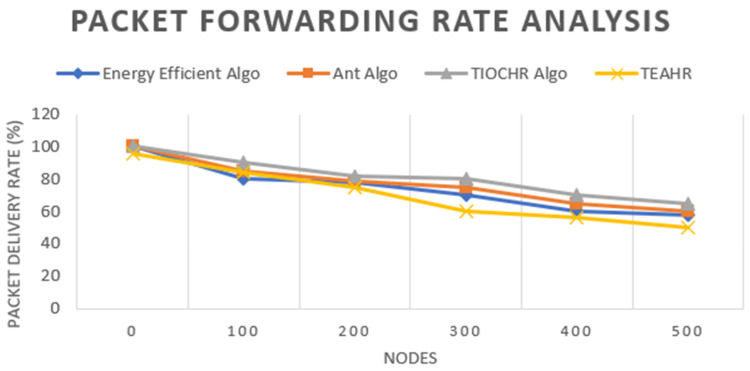
Packet forward rate analysis Energy Efficient Algo [35], Ant Algo [36], and TIOCHR Algo [34].

In Figure 12, we represent the packet forwarding rate analysis when the network grows; in this figure, all four algorithms start with high efficiency in smaller networks but begin to diverge as more nodes are introduced. The Energy Efficient and Ant algorithms show a moderate decrease in packet delivery rate, which might be acceptable in many applications. The TIOCHR algorithm performs slightly better, implying a more robust handling of packet forwarding under scaling conditions. The TEAHR algorithm stands out for its remarkable stability, indicating a robust approach to packet forwarding that is less affected by network scaling.

**Figure 13:**
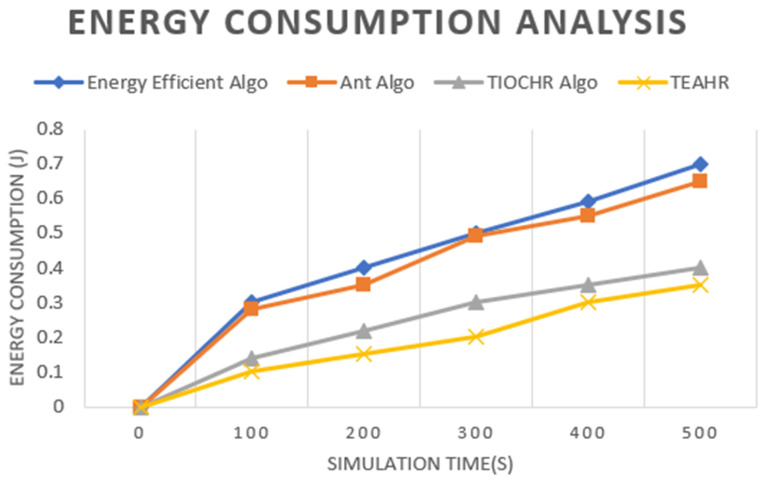
Energy consumption analysis Energy Efficient Algo [35], Ant Algo [36], and TIOCHR Algo [34].

Energy consumption plays a pivotal role over time in wireless sensor networks; in Figure 13, all algorithms increase their energy consumption over time; none of them show a decrease in energy, which might be expected as resources are consumed and tasks become more energy-intensive. The TEAHR algorithm stands out as the most energy-conserving algorithm throughout the simulation, maintains a stable packet forwarding rate, and enhances energy efficiency by around 20%. This characteristic could make it a preferred choice in scenarios where energy conservation is crucial, such as in battery-powered devices or remote sensing equipment. The initial energy efficiency of the TIOCHR algorithm may be advantageous in short-term applications, but for more extended simulations or real-time applications, its energy usage becomes less favorable.

While TEAHR offers advanced trust management and robust security features, its computational complexity may not be suitable for all types of sensor networks. TEAHR is designed for moderate-to-large-scale WSNs where security, trust, and resilience are of utmost importance. These networks typically operate in dynamic, high-risk environments, where anomalous activities, unreliable nodes, and false data injection pose significant threats. TEAHR may be suitable for high trustworthiness, resilience against anomaly activity, and hierarchical routing, making it feasible for long-term deployments. While TEAHR excels in security-sensitive applications, it introduces computational and energy overhead that may not be feasible for extremely low-power or highly dynamic networks.

## 5. Conclusions

By evaluating the consistency of sensor nodes based on their behavior and performance, trust management is a multi-pronged method that improves the safety of wireless sensor networks (WSNs). This is accomplished by analyzing the consistency of sensor nodes. Through the utilization of mathematical models and trust evaluation techniques, wireless sensor networks (WSNs) are able to design communication channels that are safe and protect themselves against threats that originate from within the network boundaries. As the state of the art in technology continues to improve, there is little question that trust management systems will continue to play an essential role in guaranteeing the security and efficiency of wireless sensor networks. Our Trusted Energy-Aware Hierarchical Routing (TEAHR) for Wireless Sensor Networks displays good performance across a variety of parameters, such as an examination of the average latency, an analysis of the packet forwarding rate, and an analysis of the energy usage. After doing these evaluations, it was discovered that the TEAHR algorithm had the lowest energy usage throughout the duration of the simulation. As a result, it was determined to be the most energy-efficient algorithm among those that were selected for comparison. The TEAHR dynamic method makes this algorithm more robust; we need to further test this by utilizing a hybrid technique; then, we can make sure that the algorithm is more resilient, energy efficient, and secure enough to detect anomalies in wireless sensor networks by maintaining Trust among the sensor’s nodes. To do this, we need to make sure that the algorithm is able to detect anomalies in wireless sensor networks. Our proposed algorithm reduces total latency by 15%, enhances energy efficiency by around 20%, and maintains a stable packet forwarding rate. This algorithm is even more potent because of the TEAHR dynamic approach.

## Figures and Tables

**Table 1:** Comparative analysis of trust models.

Feature/ Aspect	Probability Trust Model	Weighted Trust Model	Fuzzy Logic Trust Model	Game Theory-Based Trust Model	Directed Graph-Based Trust Models	Undirected Graph-Based Trust Models	TEAHR (Proposed)
**Trust Calculation Method**	Updates trust based on Bayesian inference; uses past behavior to adjust trust scores.	Aggregates various trust metrics with weighted importance factors	Uses fuzzy logic to combine various metrics; Trust is determined by fuzzy inference rules	Models trust as a game where nodes interact and adjust strategies based on outcomes	Trust is calculated by summing the weights of incoming edges from other nodes	Trust is calculated based on the average weight of connected edges	Dynamic trust updates based on multiple metrics
**Inputs Considered**	Previous trust values, successful interactions, and total interactions	Metrics like packet forwarding ratio, data trustworthiness, energy levels	Message success rate, elapsed time, correctness, fairness	The payoff, node actions, utility functions	Weights of edges in the directed graph	Weights of edges in the undirected graph	Energy, Forwarding, Consistency, Behavioral
**Handling of Uncertainty**	Incorporates prior probabilities to handle uncertainty	Handles uncertainty by adjusting weights dynamically	Fuzzy logic manages uncertainty by handling imprecise input values	Game theory models interactions under uncertainty and evolves strategies over time	Directly handles asymmetric trust relationships	Handles symmetric trust relationships	Adaptive trust thresholds, anomaly detection
**Applicability**	Suitable for scenarios with historical behavior data	Best for networks where multiple trust metrics are relevant	Ideal for networks with complex, multifaceted trust metrics	Effective in dynamic and adversarial environments where strategies evolve	Useful in networks where Trust is not reciprocal or equal between nodes	Applicable in scenarios where mutual Trust is required between nodes	Dynamic adjustment based on network conditions
**Strengths**	Adjusts well to new data, incorporates past behaviors	Flexible and adaptable with various trust metrics	Handles complex and vague data inputs effectively	Encourages cooperative behavior, handles adversarial nodes well	Effective for modeling directed trust relationships	Provides a clear model for mutual Trust in networks	High security, energy efficiency
**Weaknesses**	It can be computationally intensive, requires a lot of historical data	Requires careful selection of weights; might be sensitive to metric importance	Complexity in defining fuzzy rules and membership functions	Requires sophisticated modeling; might be complex to implement	May not handle symmetric relationships well	It may not represent asymmetric trust relationships effectively	Computationally intensive for very large-scale networks

**Table 2:** Notation description.

Notation	Description
$T_{basic}\left(i\right),E_{res}\left(i\right),E_{ref}$	The fundamental trust score of Node I is based on its residual energy relative to a reference energy level.
$T_{norm}\left(i\right),E_{res}\left(i\right),E_{max}$	Normalized trust score of nodes i relative to the maximum residual energy in the network.
$T_{weighted}\left(i\right),E_{res}\left(i\right),E_{ref},w_{1},\;w_{2},F(i)$	The weighted trust score of nodes considers energy-based Trust and additional factors like centrality or mobility.
$P_{ch}^{\prime }\left(j\right),P_{ch}\left(j\right),E_{res}\left(j\right),E_{avg\_ann}-1$	Updated CH probability for node j based on residual energy and the average residual energy of neighboring nodes.
$S\left(i,k\right),\;\beta_{1}{,\;{\beta_{2},\;\beta }_{3},\;E}_{res}\left(i\right),D\left(i,k\right),P_{ch}(i)$	Suitability metric for selecting a tentative CH based on residual energy, distance, and CH probability.
$M\left(i,j\right),\gamma 1,\;\gamma 2,\;\gamma 3\;Eres\left(j\right),\;D\left(i,j\right),C(i,j)$	A composite metric for selecting the most suitable CH based on energy, distance, and communication cost.
$T_{hreshold},network\;conditions,historical\;data$	Dynamic trust threshold that adapts based on network conditions and historical data.
$T_{new}\left(i,t\right),{\;T}_{initial},\delta ,T_{observed\;actions}(i,t)$	The new trust value for Node i at time t was adjusted based on observed actions and initial Trust.

**Table 3:** Parameters for calculating the Trust.

S.No.	Parameter	Description	Typical Values or Ranges
1	Trustworthiness (TB(x_i_))	Range from 0 (untrustworthy) to 1 (fully trustworthy), with thresholds for roles based on value.	>0.7 for Trust, <0.7 to >0.3 for partial Trust, <0.3 for untrust
2	Packet Forwarding Rate (PFR)	Part of Px_i_F() indicates trustworthiness in forwarding packets; closer to 1 is better.	Close to 1
3	Anomaly Factor (AF)	It reflects a deviation from expected behavior, part of AF(x_i_); being closer to 0 indicates normal behavior.	Close to 0
4	Residual Energy for CH Selection	Nodes with more than a specified percentage of their initial energy (e.g., >50%) are eligible for CH selection.	>50%
5	Trust Level for CH Selection	A minimum trust level (e.g., >0.7) is required for a node to be considered for CH selection.	>0.7
6	Malicious Threshold (TB(x_i_))	Nodes with a trust level below a certain threshold (e.g., <0.3) are considered malicious and blocked.	<0.3
7	Key Refresh Interval	The frequency of cryptographic key refreshes depends on security requirements (e.g., every 24 h).	Every 24 h or as needed
8	Trust Evaluation Interval	Frequency of trust evaluations, adjusted based on environment stability (e.g., every hour in stable environments).	Every hour to every 10 min
9	New Node Trust Initialization	A neutral initial trust level is assigned to new nodes until enough behavioral data are collected (e.g., 0.5).	0.5
10	Cluster Size Range	Adjustable range based on network density and area, typically 5 to 20 nodes per CH.	5 to 20 nodes per CH

**Table 4:** Multilevel trust assessment performance analysis.

Metric	Value
Computation Time (sec)	0.006
Memory Usage Before (MB)	2.60
Memory Usage After (MB)	2.53
Overhead (MB)	0.076

**Table 5:** Spoofing analysis.

Num Nodes	Num Anomaly	Anomaly Percentage	Pre Attack Excluded	Pre Attack Anomaly Excluded	Pre Attack Detection Rate	Post Attack Excluded	Post Attack Anomaly Excluded	Post Attack Detection Rate	Spoofing Success Rate
500	145	29	253	84	57.93	238	69	47.58	52.41
1000	292	29.2	508	157	53.76	487	136	46.57	53.42
2000	585	29.25	1007	307	52.47	958	258	44.10	55.89

## Data Availability

The datasets generated and/or analyzed during the current study are available from the corresponding author upon reasonable request.
